# Drink and Accidents

**Published:** 1895-04-13

**Authors:** Sydney Holland

**Affiliations:** Chairman of the Poplar Hospital for Accidents


### DRINK AND ACCIDENTS.

By the Hon. Sydney Holland, Chairman of the Poplar Hospital for Accidents.

If one wants to prove something and collects figures for that purpose, the figures can be, and generally are, manipulated to prove that "something." This is the so universal experience of the use of statistics that it has become proverbial. I am not a teetotal advocate, and I analysed the cause of the accidents at the Poplar Hospital simply as a matter of public interest, and to prove nothing, and with no idea of proving anything. Are my figures accurate? How were they obtained? Let me tell you. For the last six months our house-surgeons have used the following letters to indicate where the accident occurred: H, home; W, work,; S, street; D, due to drink or patient drunk; A, assault, not drunken; T E, teeth extracted. And these signs have been put down in the casualty book immediately on the patient being seen. Neither of the three house-surgeons are teetotal advocates, nor was there any idea of quoting these figures to prove or disprove any question with reference to the number caused by drink or any other cause. So I may claim this that these figures, as far as the Poplar Hospital accidents is concerned, testify to actual facts. All the house surgeons were surprised, and all said, "I should have thought more were due to drink," just as Mr. Forbes Fraser, Mr. Abbott, Mr. Martin, and Mr. Davies said to your itinerant ambassador. A labour leader, on the other hand, expressed no astonishment but said that times in Poplar had been too bad for much drunkenness, and that with a revival of trade there would be an increase of drunkenness. This is somewhat confirmed by our senior house surgeon's (Mr. Radford) opinion, and he has had four years' experience at Poplar. I think, however, that Mr. Escombe, of Charing Cross, has put his finger on the weak spot of my miraculous deduction. He has pointed out truly that a large number of accidents which are brought to hospitals are insignificant and trivial, and adds that if these were counted as "accidents" for the purposes of the enumerator (as they were) he would not be surprised if it worked out that only one in fifty and not one in twenty-seven were due to drink. I am in no way wedded to my deduction, and I think that Mr. Escombe's explanation is in some measure true. But insignificant and trivial injuries are "caused by drink," and to drunkards just as much as to sober people, and if I were to go through the cases, and eliminate the trivial injuries, the result would then be dependent on what I considered trivial or not. However, let us wait another six months, and see whether the six I have quoted from were exceptional or not. Meanwhile, my figures have done their work in obtaining for the Poplar Hospital a gratuitous advertisement (with so far no pecuniary result) in most papers in England.

